# Error, reproducibility and uncertainty in experiments for electrochemical energy technologies

**DOI:** 10.1038/s41467-022-34594-x

**Published:** 2022-11-11

**Authors:** Graham Smith, Edmund J. F. Dickinson

**Affiliations:** National Physical Laboratory, Hampton Road, Teddington, TW11 0LW UK

**Keywords:** Electrocatalysis, Materials for energy and catalysis, Electrochemistry, Fuel cells, Batteries

## Abstract

The authors provide a metrology-led perspective on best practice for the electrochemical characterisation of materials for electrochemical energy technologies. Such electrochemical experiments are highly sensitive, and their results are, in practice, often of uncertain quality and challenging to reproduce quantitatively.

A critical aspect of research on electrochemical energy devices, such as batteries, fuel cells and electrolysers, is the evaluation of new materials, components, or processes in electrochemical cells, either ex situ, in situ or in operation. For such experiments, rigorous experimental control and standardised methods are required to achieve reproducibility, even on standard or idealised systems such as single crystal platinum^1^. Data reported for novel materials often exhibit high (or unstated) uncertainty and often prove challenging to reproduce quantitatively. This situation is exacerbated by a lack of formally standardised methods, and practitioners with less formal training in electrochemistry being unaware of best practices. This limits trust in published metrics, with discussions on novel electrochemical systems frequently focusing on a single series of experiments performed by one researcher in one laboratory, comparing the relative performance of the novel material against a claimed state-of-the-art.

Much has been written about the broader reproducibility/replication crisis^2^ and those reading the electrochemical literature will be familiar with weakly underpinned claims of “outstanding” performance, while being aware that comparisons may be invalidated by measurement errors introduced by experimental procedures which violate best practice; such issues frequently mar otherwise exciting science in this area. The degree of concern over the quality of reported results is evidenced by the recent decision of several journals to publish explicit experimental best practices^3–5^, reporting guidelines or checklists^6–10^ and commentary^11–13^ aiming to improve the situation, including for parallel theoretical work^14^.

We write as two electrochemists who, working in a national metrology institute, have enjoyed recent exposure to metrology: the science of measurement. Metrology provides the vocabulary^15^ and mathematical tools^16^ to express confidence in measurements and the comparisons made between them. Metrological systems and frameworks for quantification underpin consistency and assurance in all measurement fields and formal metrology is an everyday consideration for practical and academic work in fields where accurate measurements are crucial; we have found it a useful framework within which to evaluate our own electrochemical work. Here, rather than pen another best practice guide, we aim, with focus on three-electrode electrochemical measurements for energy material characterisation, to summarise some advice that we hope helps those performing electrochemical experiments to:

- avoid mistakes and minimise error
- report in a manner that facilitates reproducibility
- consider and quantify uncertainty

## Minimising mistakes and error

Metrology dispenses with nebulous concepts such as performance and instead requires scientists to define a specific measurand (“the quantity intended to be measured”) along with a measurement model *(*”the mathematical relation among all quantities known to be involved in a measurement”), which converts the experimental indicators into the measurand^15^. Error is the difference between the reported value of this measurand and its unknowable true value. (Note this is not the formal definition, and the formal concepts of error and true value are not fully compatible with measurement concepts discussed in this article, but we retain it here—as is common in metrology tuition delivered by national metrology institutes—for pedagogical purposes^15^).

Mistakes (or gross errors) are those things which prevent measurements from working as intended. In electrochemistry the primary experimental indicator is often current or voltage, while the measurand might be something simple, like device voltage for a given current density, or more complex, like a catalyst’s turnover frequency. Both of these are examples of ‘method-defined measurands’, where the results need to be defined in reference to the method of measurement^17,18^ (for example, to take account of operating conditions). Robust experimental design and execution are vital to understand, quantify and minimise sources of error, and to prevent mistakes.

Contemporary electrochemical instrumentation can routinely offer a current resolution and accuracy on the order of femtoamps; however, one electron looks much like another to a potentiostat. Consequently, the practical limit on measurements of current is the scientist’s ability to unambiguously determine what causes the observed current. Crucially, they must exclude interfering processes such as modified/poisoned catalyst sites or competing reactions due to impurities.

As electrolytes are conventionally in enormous excess compared to the active heterogeneous interface, electrolyte purity requirements are very high. Note, for example, that a perfectly smooth 1 cm^2^ polycrystalline platinum electrode has on the order of 2 nmol of atoms exposed to the electrolyte, so that irreversibly adsorbing impurities present at the part per billion level (nmol mol^−1^) in the electrolyte may substantially alter the surface of the electrode. Sources of impurities at such low concentration are innumerable and must be carefully considered for each experiment; impurity origins for kinetic studies in aqueous solution have been considered broadly in the historical literature, alongside a review of standard mitigation methods^19^. Most commercial electrolytes contain impurities and the specific ‘grade’ chosen may have a large effect; for example, one study showed a three-fold decrease in the specific activity of oxygen reduction catalysts when preparing electrolytes with American Chemical Society (ACS) grade acid rather than a higher purity grade^20^. Likewise, even 99.999% pure hydrogen gas, frequently used for sparging, may contain more than the 0.2 μmol mol^−1^ of carbon monoxide permitted for fuel cell use^21^.

The most insidious impurities are those generated in situ. The use of reference electrodes with chloride-containing filling solutions should be avoided where chloride may poison catalysts^22^ or accelerate dissolution. Similarly, reactions at the counter electrode, including dissolution of the electrode itself, may result in impurities. This is sometimes overlooked when platinum counter electrodes are used to assess ‘platinum-free’ electrocatalysts, accidentally resulting in performance-enhancing contamination^23,24^; a critical discussion on this topic has recently been published^25^. Other trace impurity sources include plasticisers present in cells and gaskets, or silicates from the inappropriate use of glass when working with alkaline electrolytes^26^. To mitigate sensitivity to impurities from the environment, cleaning protocols for cells and components must be robust^27^. The use of piranha solution or similarly oxidising solution followed by boiling in Type 1 water is typical when performing aqueous electrochemistry^20^. Cleaned glassware and electrodes are also routinely stored underwater to prevent recontamination from airborne impurities.

The behaviour of electronic hardware used for electrochemical experiments should be understood and considered carefully in interpreting data^28^, recognising that the built-in complexity of commercially available digital potentiostats (otherwise advantageous!) is capable of introducing measurement artefacts or ambiguity^29,30^. While contemporary electrochemical instrumentation may have a voltage resolution of ~1 μV, its voltage measurement uncertainty is limited by other factors, and is typically on the order of 1 mV. As passing current through an electrode changes its potential, a dedicated reference electrode is often incorporated into both ex situ and, increasingly, in situ experiments to provide a stable well defined reference. Reference electrodes are typically selected from a range of well-known standardised electrode–electrolyte interfaces at which a characteristic and kinetically rapid reversible faradaic process occurs. The choice of reference electrode should be made carefully in consideration of chemical compatibility with the measurement environment^31–34^. In combination with an electronic blocking resistance, the potential of the electrode should be stable and reproducible. Unfortunately, deviation from the ideal behaviour frequently occurs. While this can often be overlooked when comparing results from identical cells, more attention is required when reporting values for comparison.

**Fig. 1:**
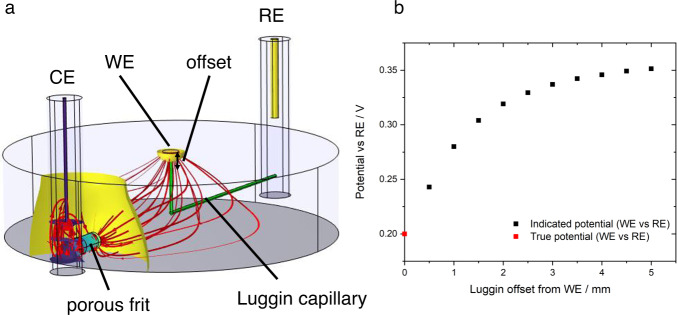
Example of error introduced by cell geometry. **a** Illustration (simulated data) of primary (resistive) current and potential distribution in a typical three-electrode cell. The main compartment is cylindrical (4 cm diameter, 1 cm height), filled with electrolyte with conductivity 1.28 S m^−1^ (0.1 M KCl(aq)). The working electrode (WE) is a 2 mm diameter disc drawing 1 mA (≈ 32 mA cm^−2^) from a faradaic process with infinitely fast kinetics and redox potential 0.2 V vs the reference electrode (RE). The counter electrode (CE) is connected to the main compartment by a porous frit; the RE is connected by a Luggin capillary (green cylinders) whose tip position is offset from the WE by a variable distance. Red lines indicate prevailing current paths; coloured surfaces indicate isopotential contours normal to the current density. **b** Plot of indicated WE vs RE potential (simulated data). As the Luggin tip is moved away from the WE surface, ohmic losses due to the WE-CE current distribution lead to variation in the indicated WE-RE potential. Appreciable error may arise on an offset length scale comparable to the WE radius.

In all cases where conversion between different electrolyte–reference electrode systems is required, junction potentials should be considered. These arise whenever there are different chemical conditions in the electrolyte at the working electrode and reference electrode interfaces. Outside highly dilute solutions, or where there are large activity differences for a reactant/product of the electrode reaction (e.g. pH for hydrogen reactions), liquid junction potentials for conventional aqueous ions have been estimated in the range <50 mV^33^. Such a deviation may nonetheless be significant when onset potentials or activities at specific potentials are being reported. The measured potential difference between the working and reference electrode also depends strongly on the geometry of the cell, so cell design is critical. Fig. 1 shows the influence of cell design on potential profiles. Ideally the reference electrode should therefore be placed close to the working electrode (noting that macroscopic electrodes may have inhomogeneous potentials). To minimise shielding of the electric field between counter and working electrode and interruption of mass transport processes, a thin Luggin-Haber capillary is often used and a small separation maintained. Understanding of shielding and edge effects is vital when reference electrodes are introduced in situ. This is especially applicable for analysis of energy devices for which constraints on cell design, due to the need to minimise electrolyte resistance and seal the cell, preclude optimal reference electrode positioning^32,35,36^.

Quantitative statements about fundamental electrochemical processes based on measured values of current and voltage inevitably rely on models of the system. Such models have assumptions that may be routinely overlooked when following experimental and analysis methods, and that may restrict their application to real-world systems. It is quite possible to make highly precise but meaningless measurements! An often-assumed condition for electrocatalyst analysis is the absence of mass transport limitation. For some reactions, such as the acidic hydrogen oxidation and hydrogen evolution reactions, this state is arguably so challenging to reach at representative conditions that it is impossible to measure true catalyst activity^11^. For example, ex situ thin-film rotating disk electrode measurements routinely fail to predict correct trends in catalyst performance in morphologically complex catalyst layers  as relevant operating conditions (e.g. meaningful current densities) are theoretically inaccessible. This topic has been extensively discussed with some authors directly criticising this technique and exploring alternatives^37,38^, and others defending the technique’s applicability for ranking catalysts if scrupulous attention is paid to experimental details^39^; yet, many reports continue to use this measurement technique blindly with no regard for its applicability. We therefore strongly urge those planning measurements to consider whether their chosen technique is capable of providing sufficient evidence to disprove their hypothesis, even if it has been widely used for similar experiments.

The correct choice of technique should be dependent upon the measurand being probed rather than simply following previous reports. The case of iR correction, where a measurement of the uncompensated resistance is used to correct the applied voltage, is a good example. When the measurand is a material property, such as intrinsic catalyst activity, the uncompensated resistance is a source of error introduced by the experimental method and it should carefully be corrected out (Fig. 1). In the case that the uncompensated resistance is intrinsic to the measurand—for instance the operating voltage of an electrolyser cell—iR compensation is inappropriate and only serves to obfuscate. Another example is the choice of ex situ (outside the operating environment), in situ (in the operating environment), and operando (during operation) measurements. While in situ or operando testing allows characterisation under conditions that are more representative of real-world use, it may also yield measurements with increased uncertainty due to the decreased possibility for fine experimental control. Depending on the intended use of the measurement, an informed compromise must be sought between how relevant and how uncertain the resulting measurement will be.

## Maximising reproducibility

**Fig. 2:**
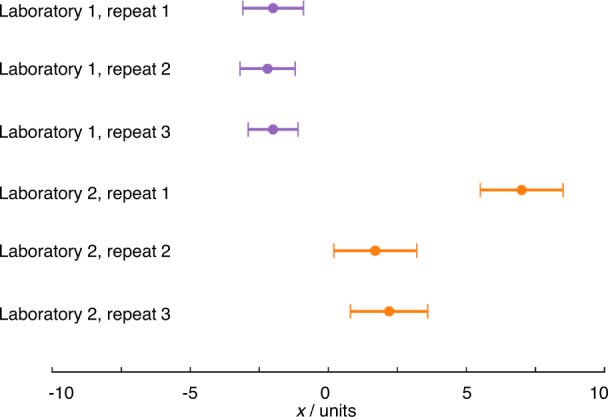
Illustration of reproducibility, repeatability and gross error. The measurements from laboratory 1 show a high degree of repeatability, while the measurements from laboratory 2 do not. Apparently, a mistake has been made in repeat 1, which will need to be excluded from any analysis and any uncertainty analysis, and/or suggests further repeat measurements should be conducted. The error bars are based on an uncertainty with coverage factor ~95% (see below) so the results from the two laboratories are different, i.e. show poor reproducibility. This may indicate differing experimental practice or that some as yet unidentified parameter is influencing the results.

Most electrochemists assess the repeatability of measurements, performing the same measurement themselves several times. Repeats, where all steps (including sample preparation, where relevant) of a measurement are carried out multiple times, are absolutely crucial for highlighting one-off mistakes (Fig. 2). Reproducibility, however, is assessed when comparing results reported by different laboratories. Many readers will be familiar with the variability in key properties reported for various systems e.g. variability in the reported electrochemically active surface area (ECSA) of commercial catalysts, which might reasonably be expected to be constant, suggesting that, in practice, the reproducibility of results cannot be taken for granted. As electrochemistry deals mostly with method-defined measurands, the measurement procedure must be standardised for results to be comparable. Variation in results therefore strongly suggests that measurements are not being performed consistently and that the information typically supplied when publishing experimental methods is insufficient to facilitate reproducibility of electrochemical measurements. Quantitative electrochemical measurements require control over a large range of parameters, many of which are easily overlooked or specified imprecisely when reporting data. An understanding of the crucial parameters and methods for their control is often institutional knowledge, held by expert electrochemists, but infrequently formalised and communicated e.g. through publication of widely adopted standards. This creates challenges to both reproducibility and the corresponding assessment of experimental quality by reviewers. The reporting standards established by various publishers (see Introduction) offer a practical response, but it is still unclear whether these will contain sufficiently granular detail to improve the situation.

Besides information typically supplied in the description of experimental methods for publication, which, at a minimum, must detail the materials, equipment and measurement methods used to generate the results, we suggest that a much more comprehensive description is often required, especially where measurements have historically poor reproducibility or the presented results differ from earlier reports. Such an expanded ‘supplementary experimental’ section would additionally include any details that could impact the results: for example, material pre-treatment, detailed electrode preparation steps, cleaning procedures, expected electrolyte and gas impurities, electrode preconditioning processes, cell geometry including electrode positions, detail of junctions between electrodes, and any other fine experimental details which might be institutional knowledge but unknown to the (now wide) readership of the electrochemical literature. In all cases any corrections and calculations used should be specified precisely and clearly justified; these may include determinations of properties of the studied system, such as ECSA, or of the environment, such as air pressure. We highlight that knowledge of the ECSA is crucial for conventional reporting of intrinsic electrocatalyst activity, but is often very challenging to measure in a reproducible manner^40,41^.

To aid reproducibility we recommend regularly calibrating experimental equipment and doing so in a way that is traceable to primary standards realising the International System of Units (SI) base units. The SI system ensures that measurement units (such as the volt) are uniform globally and invariant over time. Calibration applies to direct experimental indicators, e.g. loads and potentiostats, but equally to supporting tools such as temperature probes, balances, and flow meters. Calibration of reference electrodes is often overlooked even though variations from ideal behaviour can be significant^42^ and, as discussed above, are often the limit of accuracy on potential measurement. Sometimes reports will specify internal calibration against a known reaction (such as the onset of the hydrogen evolution reaction), but rarely detail regular comparisons to a local master electrode artefact such as a reference hydrogen electrode or explain how that artefact is traceable, e.g. through control of the filling solution concentration and measurement conditions. If reference is made to a standardised material (e.g. commercial Pt/C) the specified material should be both widely available and the results obtained should be consistent with prior reports.

Beyond calibration and reporting, the best test of reproducibility is to perform intercomparisons between laboratories, either by comparing results to identical experiments reported in the literature or, more robustly, through participation in planned intercomparisons (for example ‘round-robin’ exercises); we highlight a recent study applied to solid electrolyte characterisation as a topical example^43^. Intercomparisons are excellent at establishing the key features of an experimental method and the comparability of results obtained from different methods; moreover they provide a consensus against which other laboratories may compare themselves. However, performing repeat measurements for assessing repeatability and reproducibility cannot estimate uncertainty comprehensively, as it excludes systematic sources of uncertainty.

## Assessing measurement uncertainty

**Fig. 3:**
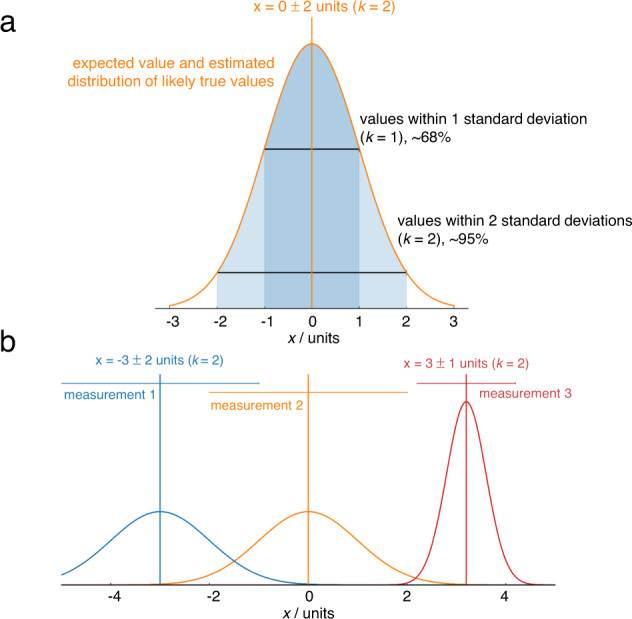
Illustrating the role of uncertainty in deciding which result is higher. **a** Complete reporting of a measurement includes the best estimate of the measurand and an uncertainty and the probability the true value falls within the uncertainty reported. Here, the percentages indicate that a normal distribution has been assumed. **b** Horizontal bars indicate 95% confidence intervals from uncertainty analysis. The confidence intervals of measurements 1 and 2 overlap when using *k* = 2, so it is not possible to say with 95% confidence that the result of the measurement 2 is higher than measurement 1, but it is possible to say this with 68% confidence, i.e. *k* = 1. Measurement 3 has a lower uncertainty, so it is possible to say with 95% confidence that the value is higher than measurement 2.

Formal uncertainty evaluation is an alien concept to most electrochemists; even the best papers (as well as our own!) typically report only the standard deviation between a few repeats. Metrological best practice dictates that reported values are stated as the combination of a best estimate of the measurand, and an interval, and a coverage factor (*k*) which gives the probability of the true value being within that interval. For example, “the turnover frequency of the electrocatalyst is 1.0 ± 0.2 s^−1^ (*k* = 2)”^16^ means that the scientist (having assumed normally distributed error) is 95% confident that the turnover frequency lies in the range 0.8–1.2 s^−1^. Reporting results in such a way makes it immediately clear whether the measurements reliably support the stated conclusions, and enables meaningful comparisons between independent results even if their uncertainties differ (Fig. 3). It also encourages honesty and self-reflection about the shortcomings of results, encouraging the development of improved experimental techniques.

**Fig. 4:**
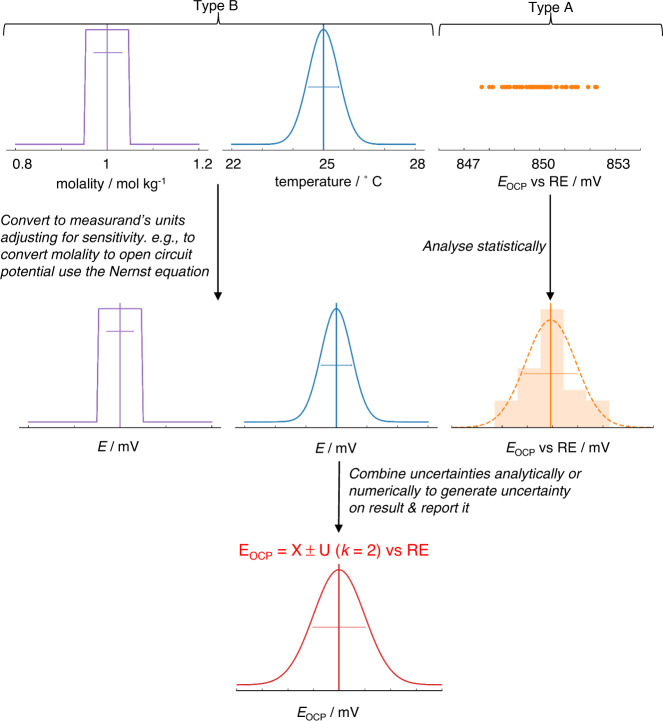
Illustration of simplified and exaggerated uncertainty evaluation on open circuit potential vs a reference electrode (RE). Possible sources of uncertainty are identified, and their standard uncertainty or probability distribution is determined by statistical analysis of repeat measurements (Type A uncertainties) or other evidence (Type B uncertainties). If required, uncertainties are then converted into the same unit as the measurand and adjusted for sensitivity, using a measurement model. Uncertainties are then combined either analytically using a standard approach or numerically to generate an overall estimate of uncertainty for the measurand (as indicated in Fig. 3a).

Constructing such a statement and performing the underlying calculations often appears daunting, not least as there are very few examples for electrochemical systems, with pH measurements being one example to have been treated thoroughly^44^. However, a standard process for uncertainty analysis exists, as briefly outlined graphically in Fig. 4. We refer the interested reader to both accessible introductory texts^45^ and detailed step-by-step guides^16,46^. The first steps in the process are to state precisely what is being measured—the measurand—and identify likely sources of uncertainty. Even this qualitative effort is often revealing. Precision in the definition of the measurand (and how it is determined from experimental indicators) clarifies the selection of measurement technique and helps to assess its appropriateness; for example, where the measurand relates only to an instantaneous property of a specific physical object, e.g. the current density of a specific fuel cell at 0.65 V following a standardised protocol, we ignore all variability in construction, device history etc. and no error is introduced by the sample. Whereas, when the measurand is a material property, such as turnover frequency of a catalyst material with a defined chemistry and preparation method, variability related to the material itself and sample preparation will often introduce substantial uncertainty in the final result. In electrochemical measurements, errors may arise from a range of sources including the measurement equipment, fluctuations in operating conditions, or variability in materials and samples. Identifying these sources leads to the design of better-quality experiments. In essence, the subsequent steps in the calculation of uncertainty quantify the uncertainty introduced by each source of error and, by using a measurement model or a sensitivity analysis (i.e. an assessment of how the results are sensitive to variability in input parameters), propagate these to arrive at a final uncertainty on the reported result.

Generally, given the historically poor understanding of uncertainty in electrochemistry, we promote increased awareness of uncertainty reporting standards and a focus on reporting measurement uncertainty with a level of detail that is appropriate to the claim made, or the scientific utilisation of the data. For example, where the primary conclusion of a paper relies on demonstrating that a material has the ‘highest ever X’ or ‘X is bigger than Y’ it is reasonable for reviewers to ask authors to quantify how confident they are in their measurement and statement. Additionally, where uncertainties are reported, even with error bars in numerical or graphical data, the method by which the uncertainty was determined should be stated, even if the method is consciously simple (e.g. “error bars indicate the sample standard deviation of *n* = 3 measurements carried out on independent electrodes”). Unfortunately, we are aware of only sporadic and incomplete efforts to create formal uncertainty budgets for electrochemical measurements of energy technologies or materials, though work is underway in our group to construct these for some exemplar systems.

Electrochemistry has undoubtedly thrived without significant interaction with formal metrology; we do not urge an abrupt revolution whereby rigorous measurements become devalued if they lack additional arcane formality. Rather, we recommend using the well-honed principles of metrology to illuminate best practice and increase transparency about the strengths and shortcomings of reported experiments. From rethinking experimental design, to participating in laboratory intercomparisons and estimating the uncertainty on key results, the application of metrological principles to electrochemistry will result in more robust science.
